# Strain-induced water dissociation on supported ultrathin oxide films

**DOI:** 10.1038/srep22853

**Published:** 2016-03-08

**Authors:** Zhenjun Song, Jing Fan, Hu Xu

**Affiliations:** 1 Department of Physics, South University of Science and Technology of China, Shenzhen, 518055, China

## Abstract

Controlling the dissociation of single water molecule on an insulating surface plays a crucial role in many catalytic reactions. In this work, we have identified the enhanced chemical reactivity of ultrathin MgO(100) films deposited on Mo(100) substrate that causes water dissociation. We reveal that the ability to split water on insulating surface closely depends on the lattice mismatch between ultrathin films and the underlying substrate, and substrate-induced in-plane tensile strain dramatically results in water dissociation on MgO(100). Three dissociative adsorption configurations of water with lower energy are predicted, and the structural transition going from molecular form to dissociative form is almost barrierless. Our results provide an effective avenue to achieve water dissociation at the single-molecule level and shed light on how to tune the chemical reactions of insulating surfaces by choosing the suitable substrates.

The interaction of water with metal oxide surfaces has attracted considerable interest due to their important promising applications in photocatalysits, electrochemistry, and sensors^1^,^2^. Understanding the mechanism of water dissociation on oxide surfaces is of fundamental interest to uncover how chemical reactions work involving water dissociation. More importantly, if we know how to control the adsorption states of water then we can selectively tune chemical reactions. Usually, hydrogen bonds play an important role in describing the structural geometry of partial dissociation of water on oxide surfaces^3^,^4^, while the intrinsic surface states are the driving force to induce water dissociation on metal oxide surfaces at various coverages^5^, especially at lower coverage.

Among oxides, MgO(100) is a good model system due to its simple structural and electronic properties to reveal chemical reactivity and catalytic activity of metal oxides. Water adsorption on MgO(100) surfaces has been intensively studied for many years, and it is well known that water will partially dissociate on MgO(100) surface at higher coverage due to strong inter-molecular hydrogen bonding^6^,^7^, while water prefers to adsorb in molecular form at lower coverage^7^. As MgO(100) is one of typical insulating surfaces, it is inactive in surface reactions and usually chemically inert towards O_2_, H_2_O, and other molecules. Recently, ultrathin MgO films deposited on metal substrates have received extensively studied^8^,^9^,^10^,^11^,^12^,^13^,^14^,^15^ due to their potential applications in catalysts. The substrate-induced enhancement of chemical reactivities has been widely reported^8^,^13^,^15^. For example, it is revealed that O_2_ can be activated to form an 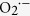 radical on MgO(100)/Mo(100) surface^15^. In addition, water adsorption on MgO(100)/Ag(100) has been studied recently, and the energy barriers for water dissociation have been effectively reduced by tuning film thickness^10^, introducing interface defects^14^ or 3d transition metal dopants^11^. Furthermore, energy differences between molecular and dissociative adsorption of water on MgO(100)/Ag(100) also decrease compared with the case for stoichiometric MgO(100) surface^10^. Unfortunately, an intact water molecule is still energetically favorable on MgO(100)/Ag(100) surface^10^,^11^,^14^. Although many efforts have been made, it remains challenging to achieve one single water molecule dissociation on MgO(100) surfaces. Therefore, it is still desirable and significant to make further efforts to strengthen the chemical activity of MgO(100) to split water.

In this work, the strain-induced water dissociation on MgO(100) is proposed theoretically, and the mechanism of water dissociation on supported ultrathin MgO(100) films is also uncovered. We demonstrate that the dissociation of a single water molecule on Mo-supproted ultrathin MgO(100) films is exothermal, and the activation barriers from molecular adsorption state of water to dissociative adsorption can be reduced significantly (nearly zero). More importantly, we provide a feasible way to modulate the adsorption states of water on supported insulating surfaces.

## Results

The calculated lattice constants for body-centered cubic (bcc) Mo and rock-salt MgO bulk are respective 3.15 Å and 4.21 Å, which are in good agreement with the experimental values^16^. Owing to the small mismatch between MgO(100) and Mo(100) surfaces, it is usually to use Mo(100) as the substrate to study ultrathin MgO(100) films. The lattice mismatch between MgO(100) and Mo(100) is 5.1%, therefore MgO ultrathin films supported on Mo(100) will slightly expand compared with their bulk position. The interlayer distance between Mo substrate and 1 ML MgO(100) is 2.10 Å, while this distance increases to 2.15 Å for 2–5 ML MgO(100). Oxygen atoms at the interface prefer to bond to surface Mo atoms, which is in line with prior results^17^.

**Figure 1:**
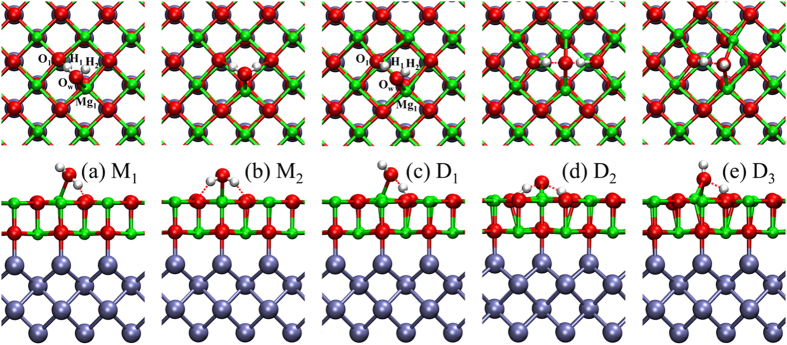
The top and side views of water adsorption on Mo(100) supported MgO(100) surfaces. Water adsorbs in molecular form with (**a**) one or (**b**) two hydrogen bonds between water and surface oxygen. (**c–e**) Water adsorbs in dissociative form.

It is well known that water molecule prefers to adsorb on the stoichiometric MgO(100) surface in molecular form at low coverage^7^. Then it will form two nearly degenerate adsorption structures with one or two hydrogen bonds between water and surface oxygen, and the corresponding adsorption energies per water are around −0.45 eV. We then study water behaviors on MgO(1–5 ML)/Mo(001) surfaces. Water will initially lands on MgO(001)/Mo(001) surfaces in the molecular form. Similarly, it is also found that water molecules have two possible adsorption configurations in molecular form with nearly degenerate adsorption energy. One molecular configuration M_1_ is that there is one strong hydrogen bond between water and surface oxygen with the distance of 1.38 Å (see Fig. 1(a)), while another molecular adsorption (M_2_) has two identical weak hydrogen bonds with the distance of around 1.68 Å (see Fig. 1(b)). The adsorption energies per water for both M_1_ and M_2_ on MgO(1–5 ML)/Mo(100) are from −0.67 eV to −0.75 eV, while the adsorption energies per water on ultrathin MgO(100) films deposited on Ag(100) are around −0.5 eV. The results indicate that molecular adsorption of water can be significantly strengthened by the Mo(100) substrate. In addition, the adsorption energy per water are almost insensitive to film thickness.

**Figure 2:**
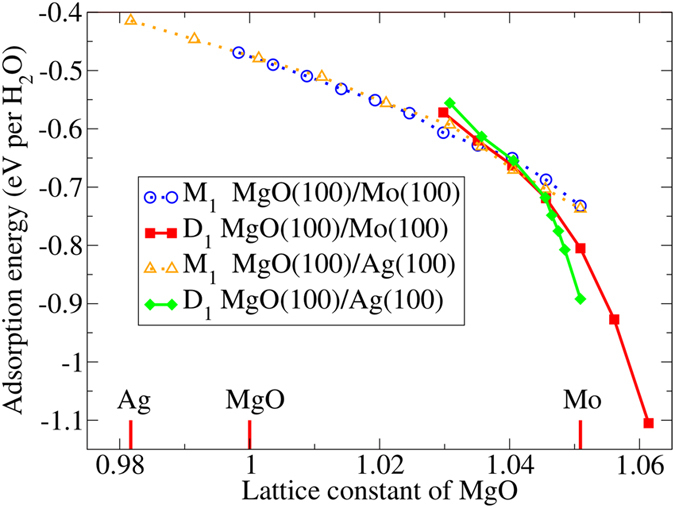
Adsorption energy per water as a function of MgO lattice constant on Ag- or Mo-supported 2 ML MgO(100) thin films. The optimized lattice constants of Ag and Mo are also marked relative to MgO.

The questions is where the adsorption energy differences for water adsorption on ultrathin MgO(100) films deposited on different metal substrates come from. It is clearly that MgO lattice is slightly contracted by 1.8% on Ag while expanded by 5.1% on Mo substrate. Is MgO lattice expansion induced by Mo substrate responsible for the enhancement of water adsorption? To verify our assumption, we have calculated the adsorption energy per water as a function of MgO lattice on MgO(2 ML)/Ag(100) and MgO(2 ML)/Mo(100) surfaces shown in Fig. 2. When ultrathin MgO(100) films deposited on Ag(100) substrate, the lattice of MgO will be shortened by 1.8%, and the corresponding adsorption energy per water is −0.41 eV. While the adsorption energy per water is −0.74 eV on Mo-supported MgO(100). If we keep the lattice parameters of MgO(100)/Mo(100) unchanged, and just replace Mo by Ag, then in this case the adsorption energy per water is −0.73 eV. Our results indicate that the adsorption energy for molecular adsorption almost linearly increases with the increasing of MgO lattice constant. In other words, the adsorption energy closely depends on the lattice parameter of MgO, while charge effect does not play an important role in water dissociation. The results definitely indicate that the expansion of MgO lattice will remarkably strengthen the interaction of water with MgO(100) surface. This is because the increment of the bond length of MgO will reduce bond strength significantly, resulting in the enhancement of their reactivity for water splitting.

Now that the interaction of water with ultrathin MgO(100) films has been greatly improved by Mo(100) substrate, it is interesting to study whether MgO(100)/Mo(100) is reactive for water dissociation. In contrast to adsorption behaviors of water on MgO(100)/Ag(100), water will easily dissociate on MgO(100)/Mo(100) surface, which implies that the ability to split water on ultrathin MgO(100) films is notably improved by Mo(100) substrate. Three possible dissociative configurations D_1_, D_2_, and D_3_ are shown in Fig. 1(c–e), respectively. The adsorption energies for molecular and dissociative adsorption for MgO(1–5 ML)/Mo(100) are listed in Table 1. From Table 1, we can find that the dissociative configurations are favored over molecular adsorption.

To uncover the dissociative mechanism of water, we systematically study the structural configurations of M_1_ and D_1_ using MgO(2 ML)/Mo(100) surface. The corresponding structural parameters and adsorption energy per water as a function of MgO lattice are listed in Table 2. The MgO lattice increases gradually from +0.0% to 5.1%, where MgO lattice with 5.1% expansion is equal to that of Mo lattice. The results clearly show that the bond length of O_w_-H_1_ in water steadily increases from 1.02 Å to 1.12 Å along with MgO lattice expansion range from 0.0% to 5.1%, where the bond length of O_w_-H_2_ in water is unaffected by the change of MgO lattice. Accordingly, the hydrogen bond between water and surface oxygen (O_1_-H_1_) gets shorter by 0.29 Å. The bond length elongation of O_w_-H_1_ and the shortening of O_1_-H_1_ indicate that water molecule tends to dissociate. In addition, from Table 2 we can clearly note that the bond length of O_w_-Mg_1_ decreases significantly with the increase of unit cell size, which implies the stronger interaction between water and surface. Furthermore, the bond length of O_1_-Mg_1_ increases by around 0.5 Å with the induced strain by Mo substrate. The angle of O_1_-Mg_1_-O_w_ (*θ*) also decreases by 10°.

As shown in Fig. 2, the slopes of adsorption energy for molecular and dissociative water behave differently. The dissociated water has a steeper slope than that of molecular one, as a result water prefers to dissociate on the MgO(100) surface when 4% interfacial strain is applied. As we know that the interfacial strain will change the lattice of ultrathin MgO films as the lattice constants of metal substrates vary. When ultrathin MgO films deposited on Mo(100) substrate, the MgO lattice is enlarged by 5.1%, so water prefers to dissociate on MgO(100)/Mo(100) surface. While MgO lattice shrinks 1.8% constrained on the Ag(100) substrate, thus water does not prefer to dissociate on this system. In fact, if we assume that Ag has the same lattice as Mo, water will also dissociate on MgO(100)/Ag(100) surface (see Fig. 2). In addition to metal substrate, the thickness of MgO(100) films also have some influence on the dissociative adsorption energy of water. For example, we can find that water in dissociative form on MgO(1–2 ML)/Mo(100) has much lower adsorption energy than that on MgO(3–5 ML)/Mo(100).

**Figure 3:**
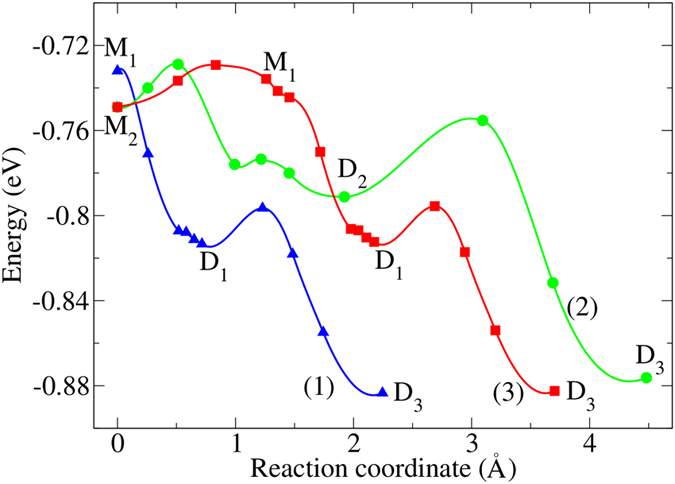
Three possible potential-energy profiles for molecular to dissociative adsorption of water on MgO(2 ML)/Mo(100): (1) M_1_ → D_1_ → D_3_, (2) M_2_ → D_2_ → D3, and (3) M_2_ → M_1_ → D_1_ → D_3_.

The corresponding reaction pathways for water dissociation on MgO(2 ML)/Mo(100) surfaces are shown in Fig. 3. Depending on the water adsorption configurations on MgO(2 ML)/Mo(100) surface, there may exist three possible water dissociation channels. For channel one (see blue line of Fig. 3), M_1_ will spontaneously transfer to D_1_ passing through a barriless pathway with the energy gain of 0.08 eV. Then D_1_ can easily transfer to D_3_ by climbing over a small barrier of 0.02 eV. D_3_ is the most energetically favorable adsorption configuration with the lowest dissociative adsorption energy of −0.88 eV. For D_3_, the O_w_H group binds to two surface Mg atoms forming two strong bonds. In addition, there exists one strong hydrogen bond between O_w_ of the dissociated water and hydrogen binding to surface oxygen. Another dissociation channel (see green line in Fig. 3) is from M_2_ to D_3_ via D_2_. It needs to overcome a very small barrier of 0.02 eV for water to dissociate initially, then it will form the meta-stable dissociative configuration of D_2_. There are two hydrogen bonds for D_2_. One hydrogen bond is that the dissociated H points to dissociated O_w_H and another one forms between H from dissociated O_w_H and surface oxygen. Afterwards, D_3_ also forms by striding over the energy barrier of 0.04 eV. Furthermore, M_2_ may transfer to M_1_ due to the small reaction barrier of 0.02 eV, then D_3_ forms going across D_1_, which is the third dissociation channel (see red line in Fig. 3). As energy barriers during water dissociation are relatively low for all the dissociation channels, there may exist multiple dissociation pathways for water on MgO(100)/Mo(100) surface. Among these, the channel one should be the most likely channel for water dissociation.

## Conclusion

In summary, we have performed a systematic study to investigate the interaction of water with Mo-supported ultrathin MgO(100) films. The understanding of how water interacts with metal oxide surfaces is important in uncovering the interfacial phenomena. The single water molecule has been successfully split on insulating surface by choosing the suitable metal substrate. The mechanism of water dissociation on MgO(100)/Mo(100) surface has been revealed. The interfacial tensile strain due to lattice mismatch will cause the expansion of MgO lattice, and 4% expansion of MgO lattice will result in the dissociation of water on supported MgO(100) surface. Our results provide an effective method to enhance the surface reactivity towards water by choosing the suitable substrate.

## Methods

Density-functional theory (DFT) calculations have been performed using Vienna ab initio simulation package (VASP)^18^,^19^ to study the water adsorption behaviors. Perdew-Burke-Ernzerhof (PBE) functional^20^ within generalized gradient approximation (GGA) is chose to describe exchange and correlation effects, as PBE functional gives the excellent description of hydrogen bonds^21^. Projector augmented wave (PAW) method^22^ is used to describe the interactions between valence and core electrons. The energy cutoff is 500 eV, and the convergence criterion on each atom during structural relaxations is less than 0.02 eV/Å. In order to avoid the inter-molecular interaction we present results using a p(4 × 4) Mo(100) surface, where the distance between the adjacent water molecules is 12.60 Å. Four atomic Mo layers with the bottom two layers fixed at their bulk positions are used to mimic the substrate, which give the converged results. One to five monolayers (ML) of MgO(100) are adopted as the ultrathin MgO films. A vacuum region of 15 Å is introduced to separate the neighbouring slabs. The (2 × 2 × 1) and (4 × 4 × 1) k-point Monkhorst-Pack samplings^23^ are used for structural relaxations and total energy calculations, respectively. The energy barriers and transition states are estimated by using the climbing image nudged elastic band (CI-NEB) method^24^.

## Additional Information

**How to cite this article**: Song, Z. *et al.* Strain-induced water dissociation on supported ultrathin oxide films. *Sci. Rep.* **6**, 22853; doi: 10.1038/srep22853 (2016).

## Figures and Tables

**Table 1:** The adsorption energies per water (unit in eV) for different water adsorption states on MgO(1–5 ML)/Mo(100) surfaces.

Thickness	M_1_	M_2_	D_1_	D_2_	D_3_
1 ML	−0.67	−0.67	−0.89	−0.86	−1.08
2 ML	−0.73	−0.75	−0.81	−0.79	−0.88
3 ML	−0.71	−0.72	−0.73	−0.69	−0.75
4 ML	−0.68	−0.68	−0.70	−0.66	−0.72
5 ML	−0.69	−0.70	−0.72	−0.67	−0.73
Bulk	−0.45	−0.45			

**Table 2:** Structural parameters and adsorption energies as a function of MgO lattice on MgO(2 ML)/Mo(100) surfaces.

	11.99 Å		12.35 Å		12.48 Å		12.60 Å	
	(+0.0%)		(+3.0%)		(+4.0%)		(+5.1%)	
	M_1_	D_1_	M_1_	D_1_	M_1_	D_1_	M_1_	D_1_
O*w*-H_1_ (Å)	1.02		1.04	1.33	1.06	1.41	1.12	1.46
O_*w*_-H_2_ (Å)	0.97		0.97	0.97	0.97	0.97	0.97	0.97
O_1_-H_1_ (Å)	1.67		1.57	1.13	1.52	1.09	1.38	1.06
O_*w*_-Mg_1_ (Å)	2.20		2.13	2.00	2.12	1.98	2.06	1.95
O_1_-Mg_1_ (Å)	2.25		2.46	2.62	2.55	2.72	2.70	2.85
*θ* (°)	71.1		66.7	61.7	64.5	60.8	60.6	58.9
E_*ad*_ (eV)	−0.48		−0.61	−0.57	−0.65	−0.66	−0.73	−0.81

*θ* is the angle of O_1_-Mg_1_-O_*w*_, and E_*ad*_ is the adsorption energy per water. The side length of p(4 × 4) Mo(100) supercell is listed in the first row, and the corresponding percentage increment comparing with MgO bulk is also listed in parentheses.
